# Primary Maxillary Bilateral Central Incisors with Two Roots

**DOI:** 10.5005/jp-journals-10005-1456

**Published:** 2017-02-27

**Authors:** Shweta S Jajoo

**Affiliations:** Assistant Professor, Department of Pedodontics and Preventive Dentistry, Bharati Vidyapeeth Deemed University Dental College and Hospital Pune, Maharashtra, India

**Keywords:** Bifurcated roots, Maxillary primary incisors, Root canal anatomy, Vertucci classification.

## Abstract

A rare case of birooted primary maxillary central incisor is reported. A 5-year-old male patient reported with carious maxillary primary anterior teeth. Radiographic examination incidentally revealed root bifurcation in primary maxillary central incisors bilaterally. This represents a rare finding. There are few cases that have reported birooted permanent incisors, but there is no case reported till date with birooted primary incisors. This report discusses the findings in the context of the available literature. The possible etiology and the need for careful assessment prior to root canal therapy are highlighted.

**How to cite this article:**

Jajoo SS. Primary Maxillary Bilateral Central Incisors with Two Roots. Int J Clin Pediatr Dent 2017;10(3):309-312.

## INTRODUCTION

Primary teeth have fewer abnormalities with respect to size and morphology as compared with permanent teeth. Rarely primary teeth have additional roots and those that do are usually primary molars and the primary canines.^1^ Some cases in the permanent dentition have reported birooted central and lateral incisors.^2^ But there is no case reported till date regarding bifurcation in primary central incisors. The present report describes a case in which the maxillary primary central incisors are birooted in mesial and distal directions.

One of the main objectives of nonsurgical endodontic treatment is the elimination of infection from root canal system and prevention of its reinfection. Thus, a prerequisite for the success of conventional endodontic procedure is the clear understanding of root canal morphology and that the entire root canal system must be shaped, disinfected, and filled.^3^ According to Ingle,^4^ one of the most important causes of endodontic treatment failure is the incomplete obturation of the root canal system. Similarly, Vertucci,^5^ DeGrood, and Cunningham^6^ reported that a considerable number of failures could be assigned to anatomical variations, such as the presence of unusual root canals.^7-10^ The purpose of the present study is to present and describe a clinical case of endodontic treatment of primary maxillary central incisors with two roots demonstrated by radiographic examination.

## CASE REPORT

**Fig. 1:**
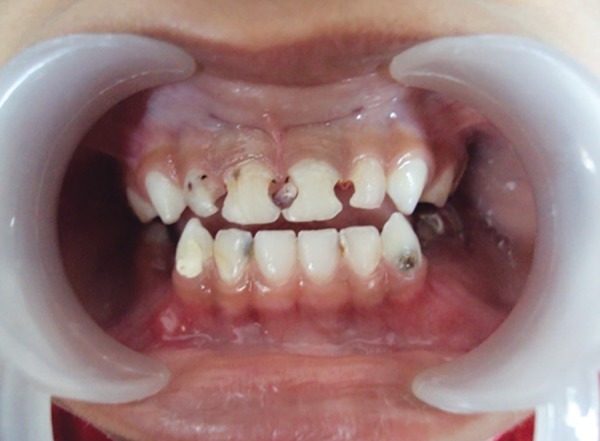
Preoperative intraoral view

A 5-year-old male patient came to the Department of Pedodontics and Preventive Dentistry, M.A. Rangoon-wala College of Dental Sciences and Research Centre, Pune, Maharashtra, India, with a chief complaint of decayed upper front teeth. The patient’s medical history was noncontributory. Patient’s mother gave a history of breastfeeding for 1 year after which the child was bottle fed for 2 years. Intraoral examination revealed primary dentition with 51, 52, 61, 62, 72, 73, 74, 75, 82, 83, 84, 85 carious teeth (Fig. 1).

**Fig. 2:**
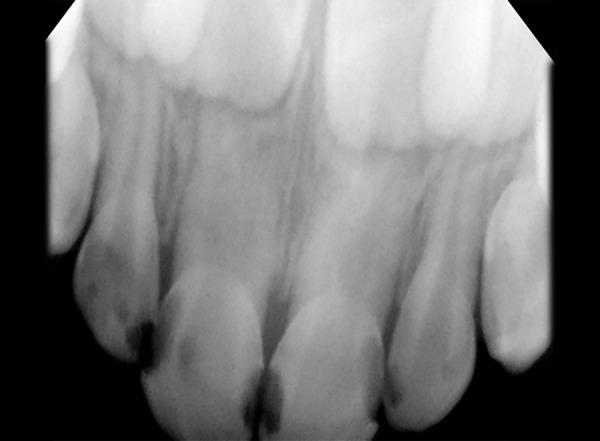
Intraoral periapical view with pulpal involvement with 51, 52, 61, 62

The intraoral periapical (IOPA) radiograph revealed pulpal involvement with 51, 52, 61, 62, 74, 75, 84, 85 (Fig. 2). In addition, when the periapical radiograph with 51, 61 was compared with normal radiograph, the pulp chamber was slightly larger in mesiodistal direction and showed the presence of two roots. The roots were bifurcated in the middle third of the root in the mesiodistal direction.

### Treatment Plan

Diet analysis, counseling, and oral prophylaxis were undertaken. The treatment planned for the pulpally involved teeth was pulpectomy, followed by composite buildup with anteriors and stainless steel crown with the posteriors. Extraction was planned for 74, 84 as the teeth were grossly decayed followed by functional space maintainer.

### Treatment Progress

The treatment plan was divided into two phases for 51, 52, 61, and 62.

- Phase 1: Endodontic phase
- Phase 2: Restorative buildup

At the first appointment, an infraorbital block was administered. A relative isolation of the upper anterior primary teeth was obtained as the child was not sufficiently cooperative.

Gross carious lesions were removed with a no. 330 round carbide steel bur (S.S. White, New Jersey, USA).

**Figs 3A and B:**
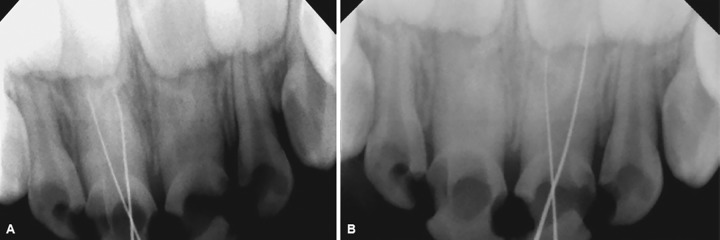
(A) Working length determination radiograph with 51; and (B) working length determination radiograph with 61

**Figs 4A and B:**
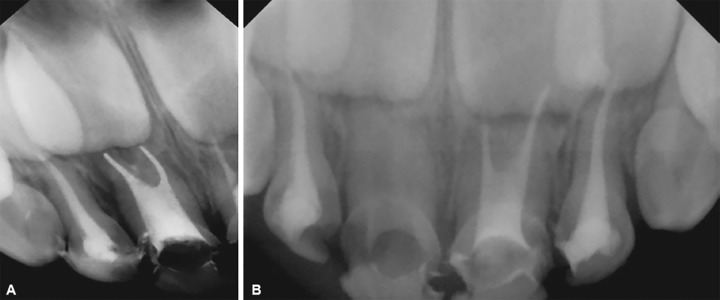
(A) Postobturation radiograph with 51; and (B) postobturation radiograph with 61, 62

The pulp chamber was opened and working length determination IOPA was taken with 52, 62 using 8 no. K-file. During canal negotiation with 51, one K-file was placed in distal canal, while the other K-file was placed in the mesial direction and working length was determined (Fig. 3). Similarly, the two canals in 61 were negotiated. The diagnostic radiovisiography was made with no. 8 and no. 10 K-files in mesial and distal canals respectively (Fig. 3A). The length obtained was 11 mm for mesial and 12 mm for distal canal. As the tooth had wide anatomical canals, the canals were prepared up to size no. 35 K-file. Filing was accompanied by frequent irrigation with 1% NaOCl followed by negative aspiration. Once the canal preparation was complete, the canals were dried with absorbent paper points. The canal space that was previously occupied by pulp was then filled with zinc oxide eugenol type I cement. A no. 35 lentulo-spiral, previously cut, was calibrated at length 11 and 12 mm and was inserted into canals to check that it could be used to correct length without binding. Once this important stage was satisfactorily completed, the lentulo-spiral was used to carry the cement into the canals in a slow-speed handpiece using forward movement. Once the cement overflowed the canals into the chamber, a check radiograph was taken; it was verified that the filling was deficient in the distal canal. The filling was adjusted and completed with the aid of no. 25 lentulo-spiral, and vertical pressure was applied with cotton balls. A new radiograph verified that the root canal filling was complete (Fig. 4). The chamber was cleaned with cotton balls.

**Fig. 5:**
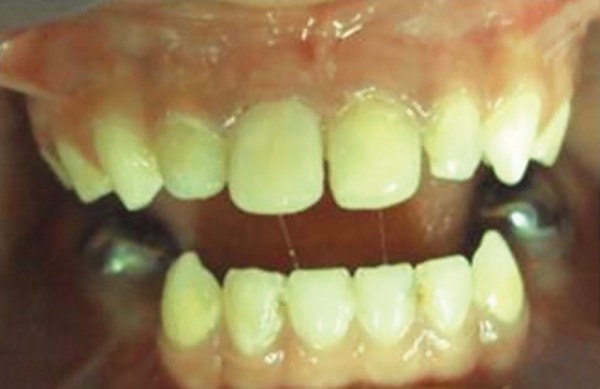
Composite builtup done with 51, 52, 61, 62

Glass-ionomer cement (type II) was placed in close proximity to canal orifice to avoid contact between zinc oxide eugenol and composite (Fig. 5). After completing the treatment, the parents were instructed to return regularly for follow-up. This follow-up was carried out every 6 months until the tooth exfoliated.

## DISCUSSION

The etiology of teeth with supernumerary roots is poorly understood. Several authors have postulated theories for the occurrence of this phenomenon. Kelly^11^ demonstrated that bifurcated roots may be related to an in-growth of Hertwig’s epithelial root sheath. Other researchers have suggested that fusion or germination may be related to the clinical presentation of supernumerary roots.^12^ The development of the roots begins after enamel and dentin formation has reached the future cementoenamel junction. The enamel organ plays an important part in root development by forming Hertwig’s epithelial root sheath, which molds the shape of roots and initiates dentin formation. There is a pronounced difference in the development of Hertwig’s epithelial root sheath in teeth with one root and in those with two or more roots.^13^ These findings suggest that in this case a defect in the dental lamina for the early stage of root formation could be an etiological factor in birooted incisors. It is also possible that an abnormality in the morphodifferentiation of the incisors may have occurred. Such abnormalities may be genetically determined or be associated with environmentally induced cellular changes.^1^

Neville et al^14^ used the term supernumerary roots in describing the development of increased number of roots in a tooth compared with a classical description in dental anatomy. When a maxillary incisor presents two roots or two root canals, conditions, such as fusion, gemination, dens in dente, and some variation in the normal development of Hertwig’s epithelial root sheath must be considered.^15,16^ In the present case, the pretreatment radiograph showed no evidence of enamel or dentinal invagination, thus making dens in dente or dens invagination unlikely causative factors.

In this case, clinical examination and pretreatment radiograph revealed a crown of normal size and shape side. Therefore, there are no chances for fusion or gemination, which results in either a single larger crown or a fused or joined crown.^16^

According to Bhaskar^13^ normal root development occurs when Hertwig’s root sheath is horizontally bent at the cementoenamel junction to narrow the cervical opening of the tooth germ. In this case report, the clinical crown has normal shape (identical to left maxillary central incisor), and it seems that during the epithelial diaphragm formation some incident caused the development of a horizontal flap of the Hertwig’s epithelial root sheath, and then the horizontal flap fused and resulted in the formation of a second root.

Endodontic success in teeth with a number of canals above that normally found requires a correct diagnosis and careful clinical radiographic inspection. Morphological variations in pulpal anatomy must be considered before treatment onset. The case presented a maxillary central incisor with two root canals, determining the developmental origin of this anatomical anomaly that appeared to have clinical significance.

## CONCLUSION

Knowledge of dental anatomy is fundamental for proper endodontic practice. When root canal treatment is performed, the clinician should be aware that both external and internal anatomy may be abnormal. Radiograph and computer tomography can help identify abnormal tooth anatomy.

## References

[1] Mochizuki K, Ohtawa Y, Kubo S, Machida Y, Yakushiji M (2001). Bifurcation, birooted primary canines: a case report. Int J Paediatr Dent.

[2] Bryant RH, Bowers DF (1982). Four birooted primary canines: report of case. ASDC J Dent Child.

[3] De Deus QD (1975). Frequency, location and direction of the lateral, secondary and accessory canals. J Endod.

[4] Ingle JI. (1985). Endodontic..

[5] Vertucci FJ (1984). Root canal anatomy of the human permanent teeth. Oral Surg Oral Med Oral Pathol.

[6] DeGrood ME, Cunningham CJ (1997). Mandibular molar with 5 canals: report of case. J Endod.

[7] de Almeida-Gomes F, de Sousa BC, dos Santos RA (2006). Unusual anatomy of mandibular premolars. Aust Endod J.

[8] de Almeida-Gomes F, Maniglia-Ferreira C, dos Santos RA (2007). Two palatal root canals in a maxillary second molar. Endod J.

[9] Sheikh-Nezami M, Mokhber N (2007). Endodontic treatment of a maxillary central incisor with three root canals. J Oral Sci.

[10] Sponchiado EC Jr, Ismail HA, Braga MR, de Carvalho FK, Simoes CA (2006). Maxillary central incisor with two root canals: a case report. J. Endod.

[11] Kelly JR (1978). Birooted primary canines. Oral Surg Oral Med Oral Pathol.

[12] Eversole LR. (1992). Dental defects. In: Clinical outline of oral pathology: diagnosis and treatment..

[13] Bhaskar SN. (1976). Orban’s oral histology and embryology..

[14] Neville BW., Damm DD., Allen CM., Bouquot JE. (2002). Oral and maxillofacial pathology..

[15] Hosomi T, Yoshikawa M, Yaoi M, Sakiyama Y, Toda T (1989). A maxillary central incisor having two root canals geminated with a supernumerary tooth. J Endod.

[16] Hatton JF, Ferrillo PJ Jr (1989). Successful treatment of a two-canalled maxillary lateral incisor. J Endod.

